# Spatial displacement of numbers on a vertical number line in spatial neglect

**DOI:** 10.3389/fnhum.2015.00240

**Published:** 2015-04-30

**Authors:** Urszula Mihulowicz, Elise Klein, Hans-Christoph Nuerk, Klaus Willmes, Hans-Otto Karnath

**Affiliations:** ^1^Division of Neuropsychology, Center of Neurology, Hertie-Institute for Clinical Brain Research, University of TübingenTübingen, Germany; ^2^Graduate School of Neural and Behavioral Sciences, International Max Planck Research SchoolTübingen, Germany; ^3^Department of Psychology, University of TübingenTübingen, Germany; ^4^IWM-KMRC Knowledge Media Research CenterTübingen, Germany; ^5^Section Neuropsychology, Department of Neurology, University Hospital RWTH Aachen UniversityAachen, Germany; ^6^LEAD Graduate School, University of TübingenTübingen, Germany

**Keywords:** numbers, space, spatial neglect, mental number line, number line estimation, human

## Abstract

Previous studies that investigated the association of numbers and space in humans came to contradictory conclusions about the spatial character of the mental number magnitude representation and about how it may be influenced by unilateral spatial neglect. The present study aimed to disentangle the debated influence of perceptual vs. representational aspects via explicit mapping of numbers onto space by applying the number line estimation paradigm with vertical orientation of stimulus lines. Thirty-five acute right-brain damaged stroke patients (6 with neglect) were asked to place two-digit numbers on vertically oriented lines with 0 marked at the bottom and 100 at the top. In contrast to the expected, nearly linear mapping in the control patient group, patients with spatial neglect overestimated the position of numbers in the lower middle range. The results corroborate spatial characteristics of the number magnitude representation. In neglect patients, this representation seems to be biased towards the ipsilesional side, independent of the physical orientation of the task stimuli.

## Introduction

There is evidence for a systematic association of numbers and space (i.e., the mental number line), but the nature of this association is still under discussion (Fias and Fischer, 2005; Aiello et al., 2012; van Dijck et al., 2012).

At the behavioral level, several space-number association effects have been observed. The “spatial-numerical association of response codes (SNARC) effect” refers to the phenomenon that responses in magnitude comparison and parity judgment tasks are faster with the left hand for relatively small numbers, whereas for relatively large numbers responses are faster with the right hand (Dehaene et al., 1993; Wood et al., 2008; for a review). These observations led to the metaphor of a horizontal, left-to-right oriented mental number line for the spatial representation of numbers (Dehaene et al., 1993). The SNARC effect has been studied for a variety of task modalities (Nuerk et al., 2005), albeit its exact source is still under debate and alternative, non-spatial explanations have been proposed (see e.g., Abrahamse et al., 2014; van Dijck et al., 2014). A different paradigm assesses the spatial properties of number magnitude representation using numbers as task-irrelevant cues (e.g., Fischer et al., 2003; Casarotti et al., 2007). In healthy participants, Fischer (2001) modified the classical line bisection task in such a way that—in one experimental condition—the lines were themselves composed of digits; or in a second condition, digits were used as flankers. A rightward bisection bias was observed when the lines consisted of digits denoting large magnitudes (8 or 9) and a leftward bias for small digits (1 or 2). When digits were used as flankers, bisection was biased towards the flanker of larger magnitude, regardless of its position (cf. De Hevia et al., 2006).

Noteworthy, spatial mapping of numbers has also been reported in the vertical dimension. In most cases, number magnitude is mapped onto space from bottom to top (e.g., Ito and Hatta, 2004; Gevers et al., 2006; Wiemers et al., 2014), however, a reversed direction has been reported as well (Hartmann et al., 2014).

Another line of evidence for spatial-numerical associations comes from observations of patients with spatial neglect (see Umiltà et al., 2009 for a review). Spatial neglect is known to affect not only afferent information from the surroundings (e.g., Karnath, 1994) but also representational space (Bisiach et al., 1981; Guariglia et al., 2013). If spatial-numerical associations exist, biases regarding the (spatial) representation of number magnitude may be expected in neurological patients suffering from neglect. Indeed, a rightward bias was observed in neglect patients in several tasks involving the mental representation of number magnitude (Hoeckner et al., 2008; Zorzi et al., 2012). In the number interval bisection task, patients with neglect showed a rightward bias similar to physical line bisection when asked to indicate a number midway between two given numbers (Zorzi et al., 2002). Specific difficulties occurred in number comparison tasks as well. In healthy participants, the distance effect (Moyer and Landauer, 1967) denotes that number magnitude comparison becomes more difficult as the numerical distance between numbers decreases. Patients with neglect respond slower and/or show larger distance effects for numbers smaller than the target (i.e., leftward from the target on a putative mental number line) (Vuilleumier et al., 2004; Zorzi et al., 2012; Klein et al., 2013; Masson et al., 2013).

However, there is also evidence that the association between numbers and space (as indicated by the SNARC effect) is flexible rather than hard-wired. First evidence for this suggestion was provided by Dehaene et al. (1993). The authors found that the reaction time advantage of left vs. right hand responses for relatively smaller numbers did not depend on the absolute magnitude of numbers, but on the relative magnitude of the respective numbers in the number range employed. Other studies investigating the influence of spatial neglect on number processing reported e.g., a specific bias in the number bisection task, but a regular SNARC effect in a parity task (Priftis et al., 2006). Bonato and colleagues (Bonato et al., 2008) replicated the digit-flanker effect in the line bisection for neglect patients, showing that bilateral numerical cues bias the bisection process depending on the numerical magnitude of the flanker. However, the authors did not find any unilateral numerical effect and suggested that explicit access to numerical magnitude is necessary for this effect to emerge (cf. Priftis et al., 2006; Zorzi et al., 2012). Also, double dissociations between a bias in number interval and in physical line bisection were observed in patients with spatial neglect (Doricchi et al., 2005; van Dijck et al., 2011). These observations argue for a more complex representation of numbers than along an oriented mental number line. Indeed, a principal component analysis performed by van Dijck et al. (2012) on different behavioral effect measures from the most common tasks used to assess the mental number line in neglect patients (number interval bisection, physical line bisection, parity judgment, and magnitude comparison) identified three factors (visuo-spatial working memory, verbal working memory, and spatial attention) with differential loadings of those tasks on the principal components. Moreover, in a recent study by Storer and Demeyere (2014) no correlation was found between number bisection bias and neglect or working memory measures. Thus, the nature of space-number associations in neglect has become even more controversial.

Previous studies investigating the influence of spatial neglect on number processing usually tested patients in the chronic state, often several months after the onset of brain damage. Instead, in the present study we decided to test acute patients to minimize the possibility that neural plasticity processes and development of compensatory strategies confound the observed relationship (cf. Karnath et al., 2011).

So far, little attention has been paid to the response format of the task: most studies investigating neglect in numerical tasks did not involve genuine spatial effects, but rather interpreted the effects in spatial terms. For instance, in the number interval bisection task a spatial representation is postulated to explain the numerical results (i.e., the numerical middle was overestimated), while the response is purely numerical. This difference may account for the dissociations between number interval and physical line bisection. The same holds for the only study, which has so far approached the vertical aspect of number representation in patients with neglect. Cappelletti and colleagues (Cappelletti et al., 2007) modified the mental number bisection paradigm modulating the semantic context by asking patients to name the number of the middle house in a row (horizontal condition) or the middle floor in a house (vertical condition). All five patients showed a bias in the horizontal number bisection condition, but only three of them in the vertical condition. A modulatory effect of spatial neglect on number processing has been shown also independently of behavioral responses by Priftis et al. (2008). In an auditory oddball task the latency of the P300 evoked potential for infrequent stimuli was larger, when small numbers were presented. This result informs about the representational aspect of number processing, however, the spatial component of this representation was not corroborated. In contrast, tasks such as the above mentioned digit-flanked line bisection involve truly spatial effects, however the involvement of a number representation is only implicit because the numerical cues are task-irrelevant.

In the present study, we thus employed the number line estimation paradigm, also called “number-to-position task” (Siegler and Opfer, 2003), i.e., a task that directly enforces building a number-space relationship by explicitly mapping numbers onto physical space. To disentangle the perceptual and representational aspects of number-space interaction, we modified the classical paradigm, so that the physical number lines to be marked were oriented vertically. In this way, despite the spatial response, left-sided spatial neglect should not affect task performance at the perceptual level. However, if the mental representation of numbers has spatial properties, a specific bias should be observed.

## Methods

### Participants

**Figure 1:**
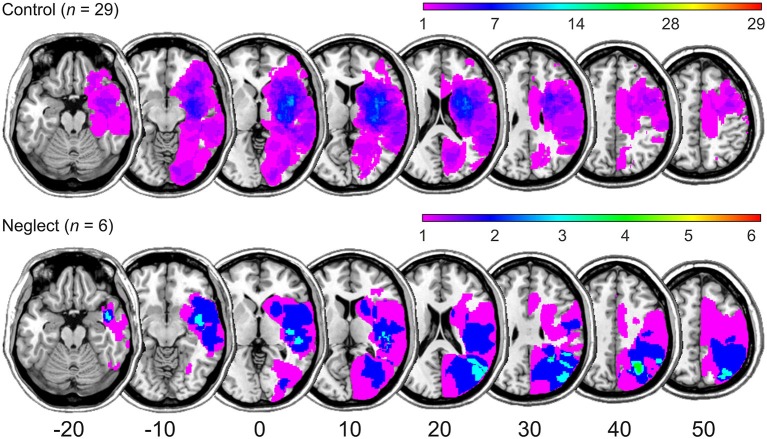
**Simple lesion-overlap for the RBD patient groups with and without neglect**. The number of overlapping lesions is color-coded with increasing frequencies from violet (*n* = 1) to red (*n* = maximum observed). MNI (Montreal Neurological Institute) coordinates of transversal sections are indicated.

Thirty-five acute stroke patients with right brain lesions participated in the study. They were consecutively admitted to the Center of Neurology at Tübingen University and fulfilled the following inclusion criteria: MR- or CT-documented cerebral stroke, max. 14 days post-stroke, no previous lesions, no other neurological or psychiatric diseases, no pronounced micro-angiopathy or white matter alterations, right-handedness, and German as their first language. Patients were considered to present with left spatial neglect (i) if they showed neglect symptoms on at least 2 out of 4 tests: letter cancellation (Weintraub and Mesulam, 1985; Center of Cancellation (CoC) > 0.083 horizontal plane), bells cancellation (Gauthier et al., 1989; CoC > 0.081 horizontal plane), horizontal line bisection (Heilman and Valenstein, 1979; rightward deviation > 14%), and a copying task (Johannsen and Karnath, 2004; > 1 error points); or (ii) if they had a CoC-value > 0.200 (horizontal plane) in at least one of the two cancellation tasks (Rorden and Karnath, 2010). Figure 1 presents the simple lesion-overlap of the two patient groups. In the control patients group mean lesion size was 30933 voxels (SD = 34951) and the largest overlap was around the insula and putamen. In the neglect patients group the largest overlap was around the occipito-parietal junction as well as middle and superior temporal gyri (mean lesion size = 93002, SD = 74762). Demographic and clinical data of the two patient groups is presented in Table 1. All patients gave their informed consent to participate. The study was conducted in accordance with the ethical standards laid down in the 1964 Declaration of Helsinki and was approved by the ethics committee of the University Clinic Tübingen.

**Table 1:** **Demographic and clinical data of the two patient groups**.

		Neglect	Control
*n*		6	29
Sex (f/m)		2/4	15/14
Age (Years)	Mean (SD)	67.7 (9.4)	60.8 (15.8)
Etiology	Infarct	4	24
	Hemorrhage	2	5
Time since lesion—examination (Days)	Mean (SD)	5.5 (1.5)	5.2 (3.0)
Education (Years)	Mean (SD)	13.3 (2.5)	11.7 (4.2)
Contralateral paresis	% present	66.7	65.5
Visual field deficit	% present	33.3	18.5
Bells cancellation (CoC)	Mean (SD)		
Horizontal		0.247 (0.100)	0.039 (0.046)
Vertical		0.071 (0.095)	0.025 (0.057)
Letter cancellation (CoC)	Mean (SD)		
Horizontal		0.203 (0.226)	0.020 (0.023)
Vertical		0.103 (0.151)	0.003 (0.027)
Copying task (Errors)	Mean (SD)	2.8 (2.5)	0.25 (0.44)
Horizontal line bisection	Mean (SD)	+6.2 (9.8)	+1.5 (4.7)
(% Deviation)
Number span backwards	Mean (SD)	3.2 (0.4)	4.1 (1.0)

### Stimuli and Procedure

Patients were asked to mark with a pencil in their right hand the position of 13 two-digit numbers on a vertically oriented line. Each of the 13 response lines was printed in black in the center of a separate DIN A4 sheet of paper and was 100 mm long and 1 mm thick. The ends of the line were marked with a horizontal bar (8 mm long). In addition, “100” was written above the top and “0” beneath the bottom bar. Sheets were aligned centrally on a table in front of the patient seated on a chair or bed. The numbers to be indicated (15, 21, 32, 39, 43, 48, 50, 53, 56, 64, 67, 78, and 81) were shown on separate DIN A4 sheets of paper (black print in the center of the sheet, digit height 9 mm). The sheets with number stimuli were presented above the sheet with the stimulus line, however for some neglect patients the sheets had to be shifted to the right to assure that the two-digit number was perceived properly. The stimuli were selected at random with the restriction that two numbers were drawn from each of the ranges: 10–30, 30–40, 40–50, 50–60, 60–70 and 70–90, plus number “50” as the numerical midpoint. Numbers with identical digits (e.g., 22) were excluded, as well as those consisting of the same digits in reversed order (e.g., if 32 was selected, 23 was excluded). The order of presentation was pseudo-randomized and the same for all participants. There was no time limit for the response and self-corrections were allowed. We made sure that patients were wearing their reading glasses, when needed.

### Analysis

Our measure of interest was the deviation of the indicated position of a number from the actual position in mm (true value—indicated value), negative differences indicating leftward bias. As a measure of general accuracy/difficulty of the task, we calculated the absolute deviation of the indicated point from the actual position in mm (|true value—indicated value|), which does not take into account the direction of bias. To identify possible differences between the patient groups, one-tailed Mann-Whitney U-tests were performed on these measures for each of the 13 positions. A Bonferroni-Holm correction was applied to control for multiple comparisons.

In addition, to better describe the performance of individual patients in the neglect patients group we performed single-case Bayesian analyses for each of the neglect patients against the group of control right brain damaged patients (Crawford and Garthwaite, 2007).

## Results

**Figure 2:**
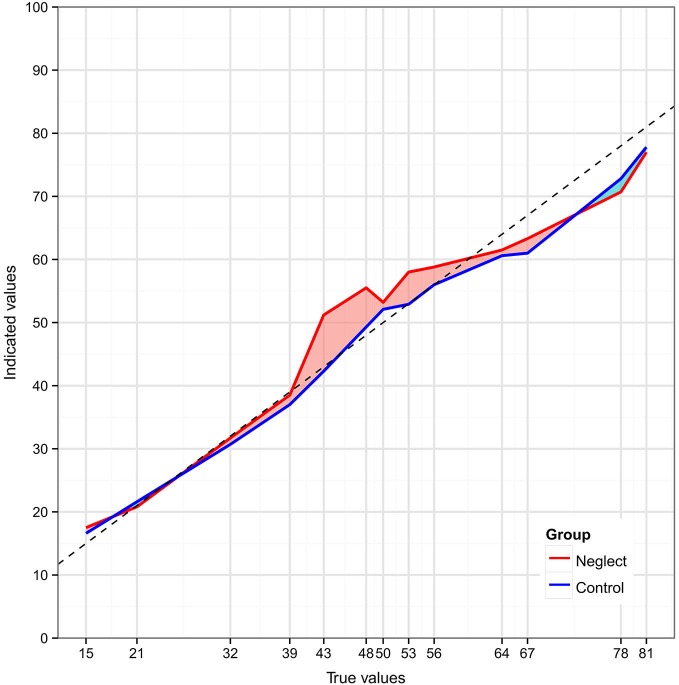
**Distribution of two-digit number placements on a vertical number line—mean values for the neglect (red) and the control patient (blue) groups**. The dashed line represents the perfect (linear) relation between number magnitude and placement.

The mean values for number positions indicated by the two patient groups are presented in Figure 2. Spatial placement of numbers in the group of control patients without neglect was nearly linear, except for slight underestimation of the position of the larger numbers. All individual estimations of the control group patients were better fitted by a linear as compared to a logarithmic function. In contrast, in the group of neglect patients the placement deviated from a linear relationship towards higher values in the lower middle range and the estimations of only 2 out of 6 patients (33.3%) were better fitted by a linear function. Response patterns of the other 4 neglect patients (66.6%) were best fitted by a logarithmic function, in which relatively higher values are assigned to smaller numbers.

In particular, the deviation score of the two groups differed at “43” (*U* = 41.5, *p* = 0.022), “48” (*U* = 21, *p* = 0.001), and “53” (*U* = 38.5, *p* = 0.016). Only at “48” the difference survived the Bonferroni-Holm correction for multiple comparisons. Interestingly, the difference between the two groups was not significant at the middle point “50” (*U* = 60, *p* = 0.127).

**Figure 3:**
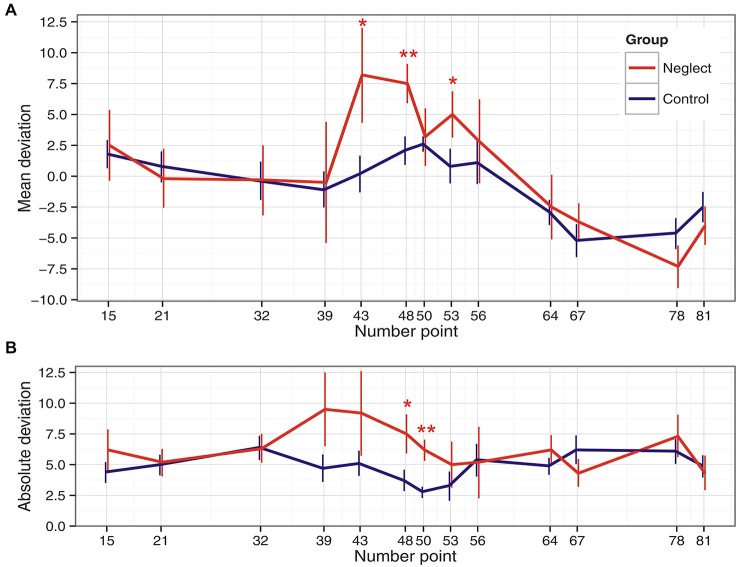
**Panel (A) depicts means and standard errors for mean deviation scores, while Panel (B) shows means and standard errors for absolute deviation scores in the two patients groups for the 13 number stimuli**. The bars represent standard errors; *difference significant at *α* = 0.05, **difference significant after Bonferroni-Holm correction for multiple comparisons.

The absolute deviation was lowest close to the ends and at the middle of the line in the control patients group. This pattern was not observed in the neglect group. Here, the deviation was largest for numbers around and below the middle of the line. Differences between the groups were found for points “48” (*U* = 33, *p* = 0.008) and “50” (*U* = 27.5, *p* = 0.004) with larger absolute differences for the neglect group. However, only point “50” remained significant after correction for multiple comparisons. The mean and absolute deviation scores of the two patients groups are depicted in Figure 3.

This pattern of results suggests that patients with neglect varied in their ability to use the midpoint of a line as a strategic anchor, if they applied the strategy of judging the part-whole proportions of the line. To better explain the data with respect to this aspect, we performed individual fittings of the estimated number lines according to the model of perceptual proportion judgment (cf. Barth and Paladino, 2011; Slusser et al., 2013, for details). Most of the individual response patterns of the control patients (23 out of 29; 79.3%) were best fitted by the two-cyclic proportion judgment model (as compared to the one-cyclic model), indicating that they were using the midpoint as a reference when assessing the position of other numbers. However, only in 50% (three out of six) of the neglect patients individual responses were best fitted by the two-cyclic model; thus half of the neglect patients were still able to use the midpoint of a line as an anchor. In sum, while the majority of right brain damaged control patients could use the number line midpoint for good performance, half of the neglect patients could not do so, in spite of the vertical presentation of the line.

When all four fitting types were compared, the majority (86.2%, 25 patients) of the control group was best fitted by the linear function that indicates optimal performance. Response patterns of the remaining four patients were best fitted by the one- or two-cyclic proportion judgment models (2 patients each). In contrast, the response patterns of 4 out of 6 neglect patients were still best fitted by a logarithmic function.

To safeguard against excessive variability in the control group in the single-case analyses, we excluded two patients whose mean scores of both mean deviation and absolute deviation were more than two standard deviations away from the control group’s mean. The single case analyses revealed a more complex pattern in the neglect patients group with several patients deviating from the control group at various points in the lower and middle number range (see Table 2 for details). However, after Bonferroni-Holm correction for multiple comparisons only two patients showed significant deviations from the control group: patient N2 at points “39”, “50”, and “53” (for all points corrected *p* < 0.001) and patient N5 at points “15” (*p* = 0.027), “39” (*p* = 0.022), “43” (*p* < 0.001), “48” (*p* = 0.012), “50”, (*p* = 0.022), “53” (*p* = 0.042), “56” (*p* = 0.027), “64” (*p* = 0.027).

**Table 2:** **Single-case analyses for each patient with neglect (N1–N6) compared against the control patients group at every measurement point (*p*-values from the Crawford and Garthwaite’s test (2007))**.

Number	N1	N2	N3	N4	N5	N6
**15**	0.095	0.087	0.179	0.420	0.003*	0.064
**21**	0.074	**0.049**	0.419	0.182	0.172	0.294
**32**	0.222	0.084	0.135	0.483	0.206	0.092
**39**	0.024	<0.001*	0.114	0.211	0.002*	0.200
**43**	0.332	0.232	0.184	0.285	<0.001*	**0.011**
**48**	0.139	0.139	0.276	0.139	0.001*	0.093
**50**	**0.044**	<0.001*	**0.044**	**0.020**	0.002*	**0.016**
**53**	0.124	<0.001*	0.189	0.489	0.007*	0.377
**56**	0.306	0.414	0.359	0.359	0.003*	0.471
**64**	0.475	0.289	0.223	0.394	0.003*	0.168
**67**	0.270	0.302	0.302	0.423	0.125	0.385
**78**	0.308	0.212	0.258	0.458	0.239	0.107
**81**	0.422	0.123	0.123	0.422	0.348	0.218

Bold - difference significant at α = 0.05; * - difference significant after Bonferroni-Holm correction.

**Figure 4:**
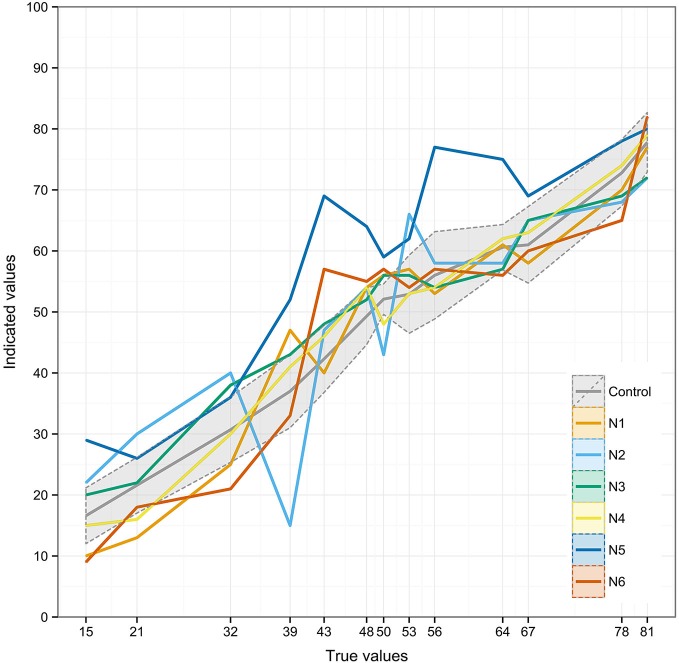
**Individual number placement patterns of the neglect patients presented against the average of the control patients group (gray line)**. The shaded area represents ±1 standard deviation from the mean of the control patients group.

Figure 4 depicts the individual number line estimation patterns of the neglect patients against the average of the control patients group. Although most of the estimated points lie within the control group’s range, the individual fittings suggest qualitatively different performances among the neglect patients.

## Discussion

The current study aimed to investigate the influence of spatial neglect on mental number-space relationships. In particular, we explored this relation using the number line estimation paradigm that involves spatial responses in a representational task for numbers. Crucially, we used vertically oriented line stimuli, allowing for disentangling the perceptual and representational aspects of number-space interaction. We observed that, whereas the distribution of number placements in the control patients group was nearly linear, the group of patients with spatial neglect indeed overestimated the position of numbers in the lower middle range close to the strategic midpoint of 50 on the number line. The current results indicate that unilateral spatial neglect can influence explicit mapping of numbers onto physical space, independent of the presence of a physical left-right dimension in the stimulus and response setting. This suggests that it is the altered mental representation of numbers that causes the observed bias, rather than perceptual properties of the task.

Neglect patients showed difficulties while estimating positions of numbers in the lower middle number range, where the absolute deviation score reflecting general processing variability of the group was largest. However, in contrast to the interpretation of Aiello et al. (2013), the difficulties did not concern small numbers in general—there was no difference between the patient groups for the smallest numbers involved, which were close to the “0” anchor. This pattern of results resembles the observations from the number comparison paradigm: when asked to indicate whether a given number is smaller or larger than a target, e.g., 5, neglect patients showed slower reaction times for numbers immediately smaller than the target, e.g., 4. (Vuilleumier et al., 2004)—and not for the smallest numbers *per se*. Further, the results of Vuilleumier and colleagues were modulated by the semantic context of the task. This emphasizes the role of task format and context in which the mental representation of numbers is invoked (cf. Dehaene et al., 1993; Fischer et al., 2010). As expected, when directionality was considered, the observed bias was towards larger numbers.

Although the effect of number displacement is significant at the group level, single-case comparisons of individual neglect patients with the control patients group only reach significance for two out of six patients after correction for multiple comparisons. Moreover, one of these patients showed a highly variable pattern over- and underestimating numbers in the middle and lower range, which can be interpreted as a general difficulty with processing numbers in this range.

On the one hand, the lack of significant differences at the individual level might be explained by low power of the test, which gets higher when results are aggregated as a group. On the other hand, the heterogeneity of the presented group of neglect patients might suggest that the effect of spatial neglect on number representation is more variable than previously assumed and individual differences may be observed. In a way, it is consistent with previous literature reporting both an influence of spatial neglect on number processing (e.g., Zorzi et al., 2002; Priftis et al., 2008; Klein et al., 2013) and its absence (e.g., Loetscher et al., 2010; Storer and Demeyere, 2014).

Overestimation in number placement can be seen as analogous to rightward bias in physical line bisection, if a left-to-right oriented mental number line is assumed (Zorzi et al., 2002). Interestingly, in our sample only one neglect patient showed a significant bias in the horizontal line bisection task. In all other patients the bisection bias was smaller than the critical 14% of the line length cut-off (Ferber and Karnath, 2001) or was even leftward of the true center. Still, despite not showing a deficit in a horizontal line bisection task, the group of neglect patients differed from the right-brain damaged control group with regard to spatial mapping of numerical information. A bias in physical line bisection has been reported to dissociate from biases in several numerical tasks, which do not explicitly involve a spatial response (e.g., van Dijck et al., 2011, 2012). The present data demonstrate that spatial neglect can influence explicit mapping of numbers in space without causing a significant bias in physical line bisection.

This finding is also related to the issue of dissociations between single measures in spatial neglect (Ferber and Karnath, 2001; Schubert and Spatt, 2001; see Vuilleumier, 2013 for a review). The results of previous studies on neglect and number processing may as well differ due to different diagnostic criteria for neglect. However, all of the neglect patients in our sample were selected for showing a significant deficit in cancellation tasks, which assess predominantly visual search and attentional orienting aspects of spatial neglect. Results of the present study can thus be interpreted in this particular context.

In addition, a general effect of neglect severity on the deviation in the experimental task might be expected, as shown in the cued line bisection paradigm (Bonato et al., 2008). Regression analyses performed for all patients revealed a significant correlation between mean CoC-scores and deviation in the experimental task only for point “32” in the neglect patients group (Bonferroni-Holm corrected for multiple comparisons). No significant correlations were observed between the bias in the experimental task and the % deviation score in horizontal line bisection. This pattern of results suggests that a general effect of neglect severity cannot be excluded and most probably it is particularly related to numbers in the lower range. However, no strong claims can be made about the presence of such a relationship. The absence of such a relationship has been reported e.g., in the number bisection paradigm (Aiello et al., 2012).

It should be noted that aside from the spatial neglect in the horizontal direction, neurological patients may also exhibit spatial bias in the vertical direction. This condition is referred to as “altitudinal neglect” (Rapcsak et al., 1988; Halligan and Marshall, 1989; Pitzalis et al., 1997), or “diagonal neglect” (Mark and Monson, 1997) when in combination with horizontal bias. To explore the possibility that altitudinal neglect influenced patients’ performance on the vertically oriented number line task, we computed the mean CoC-values for all neglect patients in the vertical dimension based on the letter- and bells cancellation tests. There was no significant difference between the neglect and control patients groups regarding the vertical CoC-values and no correlation between the vertical CoC-values and the two deviation scores at any measurement point (Bonferroni-Holm corrected for multiple comparisons). The bias in the vertical number line estimation task thus cannot be attributed to a general vertical bias in the neglect patients.

Interestingly, the pattern of patients’ number placements seems to reflect general deficits in spatial processing, as well. The vast majority of control patients were best fitted by the linear model indicating optimal performance. In contrast, only one third of the neglect patients showed a linear pattern as compared to the better fitting logarithmic relationship. In addition, we performed an analysis of individual fittings with the proportion judgment model (Barth and Paladino, 2011). Reconciling the traditional debate about linear vs. logarithmic representation of numbers, this procedure assesses the number to space placements in terms of perceptual judgment of proportions and informs rather about strategies used to estimate positions on a number line. In particular, we examined if the individual number placements are better fitted with a simple one-cyclic model, or with a two-cyclic model, which assumes the use of an additional strategic anchor in the middle of the line. The results pointed out that the majority of control patients used the midpoint of the line as an anchor. In contrast, only in half of the neglect patient group the individual estimation pattern was fitted best by a model assuming the use of the line’s midpoint. However, the bias observed in the neglect patients group cannot be taken solely as a consequence of patients’ inability to judge the midpoint of the line; in contrast, the response patterns of patients identified in additional single case analyses as showing the largest bias were best fitted by the two-cyclic model, suggesting that they did use the midpoint as an anchor.

General variability in line bisection can be interpreted in terms of an extended “indifference zone”, assuming that the bisection bias is caused by an increased Weber fraction (Marshall and Halligan, 1989). For a large dataset Bonato et al. (2008) provided evidence that increased variability in line bisection is related to neglect severity and not brain damage only. In the present study we observed larger inter-individual variability in the group of left neglect patients as measured by the absolute deviation score. However, with only one presentation of each stimulus point the intra-individual reliability was not examined, so we cannot extend the findings from the line bisection paradigm to number line estimation. This issue should thus be investigated in future studies.

Another aspect worth investigating in the future is, whether the observed bias in the spatial-numerical task could be modulated by techniques used to alleviate spatial neglect. Such positive effects have previously been shown for the numerical bias in the number interval bisection tasks by applying prism adaptation (Rossetti et al., 2004) and optokinetic stimulation (Priftis et al., 2012).

In sum, the results at a group level indicate that spatial neglect may influence the representation of numbers and their mapping onto physical space. The altered mental representation of numbers is independent of the physical left-right dimension. Thus, our observations argue for a spatial component in the representation of numbers. The single-case comparisons suggest that the effect is stronger for some neglect patients than for others. Although patterns of line estimation do not implicate exactly the same mental representation (Barth and Paladino, 2011; Chesney and Matthews, 2013; Huber et al., 2014), the bias towards larger numbers supports the notion of directionality of this representation, at least in western reading cultures.

## Conflict of Interest Statement

The authors declare that the research was conducted in the absence of any commercial or financial relationships that could be construed as a potential conflict of interest.
